# Survival analysis of neonatal mortality in Ghana using three population-based surveys

**DOI:** 10.1186/s13690-021-00773-3

**Published:** 2022-01-07

**Authors:** David Teye Doku

**Affiliations:** 1 Department of Population and Health, University of Cape Coast, Cape Coast, Ghana; 2 Directorate of Research, Innovation and Consultancy (DRIC), University of Cape Coast, Cape Coast, Ghana

**Keywords:** Neonatal mortality, Survival analysis, Ghana

## Abstract

**Background:**

Neonatal mortality in many low-and middle-income countries (LMICs) remains high despite global efforts at addressing this challenge. Tackling neonatal death in LMICs is further complicated by lack of reliable data from individual countries in the region to inform effective context specific interventions. This study investigates the probability of neonatal survival and socio-demographic risk factors of neonatal mortality in Ghana.

**Methods:**

Pooled data from three population-based surveys (*N* = 12,148) were analysed using multivariable Cox Proportional Hazards regression models.

**Results:**

The risk of dying within the first 28 days of life was highest in the first week of life (early neonatal period), it then decreases sharply around the middle of the second week of life and remains low over the late neonatal period. Adjusted hazard ratios (HRs) showed that: rural residency (HR = 1.80, 95% CI: 1.15-2.75); birth order 2-3 (HR = 1.63, 95% CI: 1.10-2.42); birth order ≥7 (HR = 1.89, 95% CI: 1.07-3.33) increased the risk of neonatal death. Additionally, children born to women who were obese had higher risk of neonatal death (HR = 1.69, CI: 1.12-2.56) compared with those of women with optimal weight. Disparities in the risk of neonatal death by geographical regions were also found.

**Conclusion:**

The risk of neonatal mortality is highest during the first week of life and it is socio-demographically patterned. The findings emphasise the need to tackle socio-demographic risk factors of neonatal mortality in order to achieve the Sustainable Development Goal 3, which is aimed at reducing neonatal mortality to 12 per 1000 live births by the year 2030.

## Background

Child mortality has decreased globally over the past one and half decades. However, the levels in most low- and middle-income countries (LMICs), particularly, in Sub-Saharan Africa is unacceptably high. The phenomenon constitutes a global public health challenge and accounts for inequalities in health between and within high income countries and low- and middle-income countries (LMICs) [1]. In 2015, 5.9 million died before their fifth birthday [2, 3], and 2.9 million of these were neonatal mortality, babies who die within the first 28 days of life, thus neonatal mortality constitutes the largest proportion of under-five mortality. About four million babies die within the new born or neonatal period and nearly all of these deaths occur mainly in LMICs [1–3]. In these countries, new born deaths account for 41% of all child deaths.

Public health interventions undertaken since 1990 to reduce under-five mortality have been mainly driven by the Millennium Development Goal (MDG) 4, which aimed at a two-thirds reduction in mortality risks of children under-five between 1990 and 2015. Consequently, many LMICs have reduced the proportion of children who died before their fifth birthdays. Despite the success achieved through the MDGs over the past one and half decades, neonatal mortality figures are still unacceptably high in most LMICs. With a few exceptions, Sub-Saharan Africa is among the regions, which did not meet the MGDs targets. Over all, among the LMICs, Sub-Saharan African countries have the largest burden of neonatal mortality.

Improvement in neonatal, infant and child mortality is an important public health goal of the government of Ghana. Over the past one and half decades this goal has led to a significant reduction in the overall child mortality in the country [4]. However, Ghana is one of the countries, which could not meet the MDG 4, of reducing by two thirds the under-five mortality between 1990 and 2015 [4]. In particular, the proportion of neonatal mortality within the child mortality remains high [4]. The most recent national representative survey conducted in 2014 suggests that among births 5 year preceding the survey, neonatal mortality rate is 29 per 1000 livebirth and this constitutes 2.2 folds of the post-neonatal mortality rate [4]. To make any progress toward the realisation of the Sustainable Development Goal (SDG) 3 target 3.2, which aims at reducing neonatal mortality to 12 per 1000 live births by the years 2030 [5], it is necessary to understand the risk factors for neonatal death. Quality data that provide country specific analysis of the situation to facilitate contextual interventions are critical.

Analysis of neonatal mortality in Sub-Saharan Africa including Ghana using population level data is rare. Only a handful of studies have conducted analysis of neonatal deaths in Ghana. Kayode, et al. [6] investigated the individual and community level factors influencing neonatal death in Ghana using surveys conducted in 2003 and 2008. However, studies that will increase our understanding of the time-to-event perspective of the phenomenon are essential for monitoring the impact of public health interventions geared towards reducing the burden of neonatal mortality, especially targeted interventions at critical time points during the neonatal period are essential. This study utilised pooled data from three nationally representative surveys to investigate the socio-demographic risk factors of neonatal mortality in Ghana.

## Methods

### Data source

This study used pooled nationally representative data from three cross-sectional surveys conducted in 2003, 2008 and 2014 in the Ghana version of the Demographic and Health Surveys (DHS), in order to ensure enough power, for the purpose of neonatal mortality risk analysis. The DHS uses a standardized questionnaire and methodology for data collection in order to facilitate international comparison in LMICs. The DHS have been conducted in nearly all LMICs since early 1990s. The Surveys have generated high quality data on significant demographic, economic, social and health for low- and middle-income countries and have been used in high quality research, which have contributed to the literature from these regions (http://dhsprogram.com/Publications/Journal-Articles-by-Journal.cfm). All the three surveys data analysed in this study were collected using two stage sampling approach based on the 2000 (for the 2003 and 2008 surveys) and 2010 (for the 2014 survey) Ghana Population and Housing Censuses to produce separate estimates for key indicators for each of the ten regions in Ghana. Sample clusters were selected from an updated master sampling frame constructed from the Ghana Population and Housing Censuses in the first stage of the sampling. The clusters were selected using systematic sampling with probability proportional to the population size. The second stage of selection involved systematic sampling of 30 of the households listed in each cluster. This was done to ensure adequate numbers of completed individual interviews to provide estimates for key indicators with acceptable precision and to provide a sample large enough to identify adequate numbers of under-five deaths to provide data on causes of death. Details of the DHS is published elsewhere (http://www.dhsprogram.com/data/data-collection.cfm). The outcome of singleton livebirths for each woman within the 5 years preceding the survey was considered in this study. The survey documented detailed information on the birth histories on livebirths for each woman in the reproductive age (15-49 years). This includes, the month and year of each birth, any multiple pregnancies, whether child was alive or dead, current age and age of death (if dead). Age at death was recorded in days if the child dies within 1 month of birth. Ethical clearance for the Ghana surveys was obtained from the Ghana Health Service Research Ethics Committee in Accra and permission to analyse the data was obtained from Measure DHS. Data for the study are publicly available and can be requested from Measure DHS through https://dhsprogram.com/data/available-datasets.cfm.

### Variables

The outcome variable for this study was neonatal death, which is defined as death of a live born within the first 28 days of life in accordance with the WHO definition of neonatal death. The independent variables used in this study are; maternal age (categorised in years as < 25, 25-29, 30-34, 35-39, 40-44, and 45-49), place of residence (categorised as rural urban) and body mass index (BMI). BMI was computed based on two anthropometric measurements (weight and height) taken during the surveys. It was calculated by dividing body weight (kg) by height squared (m^2^) and categorised into; underweight< 18.5 kg/m^2^, normal weight = 18.5–24.9 kg/m^2^, overweight =25.0–29.9 kg/ m^2^ and obese > = 30.0 kg/m^2^. The rest of the independent variables are: birth order (categorised as first birth, second or third birth, 4th - 6th birth, > 6th birth), maternal education (categorised as no education, primary, secondary or higher) and wealth index quintile. The wealth index quintile is a composite measure of a household’s cumulative living standard based on ownership of specified assets generated from principal components analysis and categorised into quintiles as poorest, poorer, middle, richer and richest [7].

### Statistical analysis

DHS conducted editing and imputation of missing procedures before the data were released. For records for which days of birth were missing, 15th day of the month was imputed to estimate the survival time in days. We used Cox Proportional hazard regression to estimate the hazard ratios (HRs) with their 95% confidence intervals (CIs) for neonatal mortality, which is given by *h*(*t*) = *h*_0_(*t*) exp[*b*_1_*x*_1_], where *x*_1_ is the covariate to the hazard *h*(*t*) at time *t* [8]. Given follow-up time as t_1_, t_2_, t_3_, t_4_, … t_k,_ where k is the number of patients/participants. In this study, the follow-up time for each neonate is the period from birth to death or the date of the survey, whichever occurs first. The assumption is that each event (outcome) occurs independently, hence the probability of no event (survival) between each time point is multiplied together to obtain the cumulative survival probability. The probability of being alive at time *t*_*j*_, *S*(*t*_*j*_), is calculated from *S*(*t*_*j* − 1_) the probability of being alive at *t*_*j* − 1_, *n*_*j*_ the number of patients alive just before *t*_*j*_, and *d*_*j*_ the number of events at *t*_*j*_, is given by the formula.

$$ S\left({t}_j\right)=S\left({t}_j-1\right)\left(1-\frac{d_j}{n_j}\right) $$

where t_0_ = 0 and S(0) =1. The estimated probability function is a step which changes values at each time of the event since S(t) is a constant variable [8, 9]. Adjusted hazard ratios for neonatal death were calculated for the total sample adjusting for each of the independent variables. The survey commands in Stata were used to account for the effect of the multi-stage cluster sampling on the estimates. The proportionality assumption of the multivariate survival model was tested using the Schoenfeld residuals diagnostic test and graphically examined with log (−log Survival) curves. These tests confirmed the adequacy of the multivariable model. Smoothed hazard functions stratified by rural-urban and BMI were used to investigate the differences in neonatal mortality rates. All statistical analysis were conducted using STATA/SE 14.0.

## Results

This study used high quality data pooled from three population-based studies conducted in 2003, 2008 and 2014 to investigate socio-demographic risk factors of neonatal mortality in Ghana. The weighted distribution of the independent variables and the pooled value for the three surveys are presented in Table 1. The pooled sample resulted in 12,148 respondents for this study. Table 1 also shows the distribution of neonatal death by the background characteristics. Women aged 25-29 years formed the largest proportion (26.3%) of the sample and those within the last 5 years of their reproductive age (45-49) were the smallest group (3.1%) by age categorisation. Majority (60.5%) of the respondents were in urban areas which is a reflection of the growing urbanisation in most LMICs including Ghana. Births within 5 years of the surveys were mainly in the birth order one (37.3%) and as expected the least proportion of births were in birth order seven or more (9.3%). A third of the women had no formal education, 22.1% had primary education and 45.4% had secondary education or higher. One-fifth of the women could be described as poorest while 16.1% were in the richest category by wealth index. Also, a third of the women were either overweight (BMI = 25.0–29.9) or obese (BMI > =30.0) while 6.8% were underweight (BMI < 18.5). The Greater Accra region, which is the region in which the capital city (Accra) is located, had the lowest sample in this study (2.4%) while the Northern region had the highest proportion (15.8%).

**Table 1:** Association of maternal characteristics and neonatal mortality in Ghana

Variable	N (%)	Neonatal deaths	Adjusted hazard ratios^a^
**Maternal age**			
< 25	2604 (22.3%)	21.2%	1.00
25-29	3071 (26.3%)	19.5%	0.78 (0.49-1.24)
30-34	2646 (22.7%)	23.9%	1.32 (0.81-2.14)
35-39	2052 (17.6%)	19.5%	1.18 (0.69-2.04)
40-44	951 (8.1%)	11.2%	1.18 (0.56-2.47)
45-49	356 (3.1%)	4.7%	1.51 (0.61-3.67)
**Place of residence**			
Urban	7070 (60.5%)	36.4%	1.00
Rural	4611 (39.5%)	63.6%	1.80 (1.18-2.75)
**Birth order**			
2-3	4353 (37.3%)	28.1%	1.00
First	2843 (24.3%)	31.4%	1.63 (1.10-2.42)
4-6	3403 (29.1%)	25.7%	1.01 (0.67-1.53)
≥ 7	1081 (9.3%)	14.8%	1.89 (1.07-3.33)
**Maternal education**			
No education	3807 (32.6%)	31.7%	1.00
Primary education	2576 (22.1%)	24.9%	1.27 (0.85-1.89)
Secondary or Higher Education	5298 (45.4%)	43.5%	0.88 (0.59-1.34)
**Wealth index**			
Poorest	2817 (24.1%)	23.1%	1.00
Poorer	2541 (21.8%)	19.0%	0.84 (0.52-1.37)
Middle	2277 (19.5%)	21.1%	1.37 (0.83-2.26)
Richer	2165 (18.5%)	17.8%	1.26 (0.67-2.36)
Richest	1880 (16.1%)	19.0%	1.52 (0.76-3.06)
**BMI**			
Optimum weight	5532 (61.0%)	53.8%	1.00
Underweight	614 (6.8%)	6.8%	1.35 (0.91-2.00)
Overweight	1847 (20.4%)	22.9%	1.07 (0.61-1.89)
Obese	1077 (11.9%)	16.5%	1.69 (1.12-2.56)
**Region**			
Greater Accra	1026 (2.4%)	7.7%	1.00
Central	1020 (8.4%)	9.5%	1.60 (0.89-2.91)
Western	1146 (9.4%)	8.3%	1.42 (0.78-2.59)
Volta	949 (7.8%)	9.5%	2.04 (1.11-3.76)
Eastern	1070 (8.8%)	10.1%	1.79 (1.01-3.14)
Ashanti	1558 (12.8%)	26.6%	2.41 (1.40-4.17)
Brong Ahafo	1312 (10.8%)	10.1%	1.78 (0.96-3.31)
Northern	1920 (15.8%)	11.2%	1.40 (0.79-2.50)
Upper East	1103 (9.1%)	3.5%	1.20 (0.60-2.39)
Upper West	1044 (8.6%)	4.1%	1.98 (1.03-3.81)
**Pooled sample**	12,148 (100.0%)	100%	

^a^Estimates adjusted for all other factors in the table

Adjusted hazard ratios (HRs) investigating the relationship between neonatal mortality and key characteristics of the sample are presented in the fourth column of Table 1. Rural residence (HR = 1.80, 95%CI: 1.15-2.75) increased the risk of neonatal death compared with urban residence; birth order 2-3 (HR = 1.63, 95% CI: 1.10-2.42) or having birth order seven or more (HR = 1.89, 95% CI: 1.07-3.33) increased the risk of neonatal death compared with women who were nulliparous; and children born to women who were obese had higher risk of neonatal death (HR = 1.69, 95% CI: 1.12-2.56) compared with those of women with optimal weight. Differences in neonatal mortality were also found by regions such that Volta (HR = 2.04, 95% CI: 1.11-3.76), Eastern (HR = 1.79, 95% CI: 1.01-3.14) Ashanti (HR = 2.40, 95% CI: 1.40-4.17) and Upper West (HR = 1.98, 95% CI: 1.03-3.81) regions had higher risk of neonatal death compared to those in the Greater Accra region. Furthermore, maternal age, primary school attainment (compared to no education), wealth index, underweight, and those in the Central, Western, Brong Ahafo, Northern and Upper East regions (compared to those in the Greater Accra region) had higher risk of neonatal death, although these estimates did not reach statistically significant level (*p* < 0.05).

**Fig. 1:**
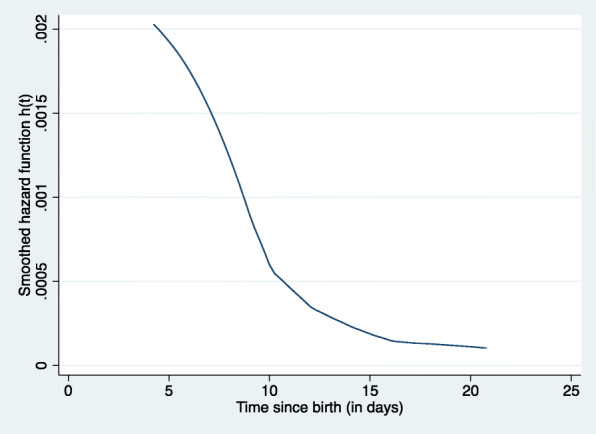
Daily hazard of death for neonates during the first month of life in Ghana from 2003 to 2014

**Fig. 2:**
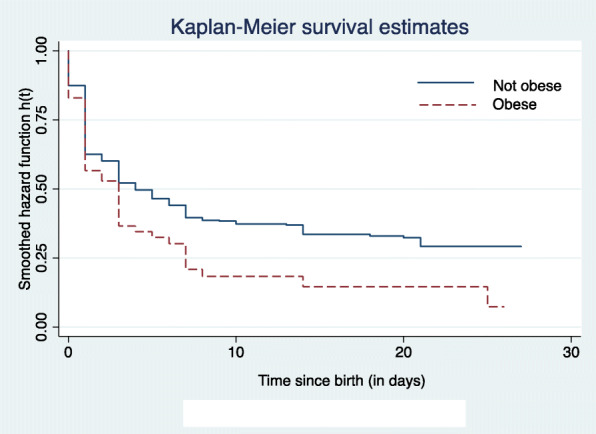
Daily survival estimate for neonates in Ghana from 2003 to 2014 by maternal body mass index

**Fig. 3:**
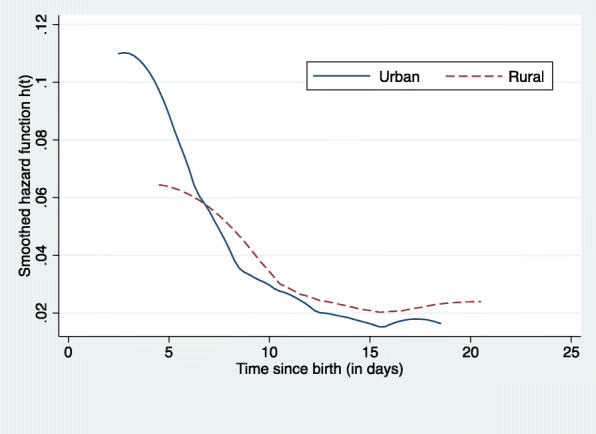
Daily hazard of death for neonates during the first month of life in Ghana from 2003 to 2014 by place of residence

Figure 1 shows the daily hazard of neonatal mortality over time. The risk of dying within the first 28 days of life was highest in the first week of life (early neonatal), it then decreases sharply around the middle of the second week of life and then remains low over the late neonatal period. Neonatal survival among obese and non-obese women is presented in Fig. 2. The risk of dying during the first 28 days of life was high for both groups in the early neonatal period and no differences were found between the two groups during this period. The probability of surviving then sharply falls among obese women creating differences between the two groups throughout the neonatal period. Figure 3 shows the smoothed hazard function for 28 days by the place of residence. The risk of neonatal death was higher for those in urban residence during the first week of life (early neonatal period). However, the risk becomes high for rural folks during the second week while no difference was observed during the late neonatal period (Fig. 3).

## Discussion

The study suggests that the risk of dying within the first 28 days of life was highest in the first week of life (early neonatal period), it then decreases sharply around the middle of the second week of life and then remains low over the late neonatal period. Rural residency, birth order 2-3 and seven or more birth order, and maternal obesity are risk factors for neonatal death among children in Ghana. In addition, regional differences in the risk of dying during the first 28 days of life were found such that Volta, Eastern, Ashanti and Upper West regions had higher risk of neonatal death compared to the Greater Accra region. Furthermore, the finding suggests that these risks of neonatal mortality were highest during the first week of life.

Consistent with previous findings, older maternal age was found to be a risk factor for neonatal mortality in this study, albeit not statistically significant [10]. Kozuki, et al. (2013) [11] in a meta-analysis investigating the associations of parity and maternal age with small-for-gestational-age, preterm delivery and neonatal and infant mortality, found that women with birth 2-3 nulliparous who were aged < 18, compared with women who had parity 1-2 and aged 18- < 35 years had the highest odds of preterm and neonatal mortality. Increased risk of neonatal death observed among those with birth order seven or more also supports the aforementioned literature. The relationships between neonatal mortality and both age and birth order have biological plausibility. More so, some previous studies have found differences in maternal health utilisation by maternal experience, in terms of age and birth order which could explain partly the association found here [12–14].

A number of earlier studies have documented disparities in the important child health outcomes including child mortality and nutrition between rural and urban setting in the LMICs [15–19]. Most of these studies have found differences in maternal and child related outcomes to the disadvantage of rural dwellers [16, 17] but a couple of them also found differences to the peril of urban populations [20]. Several factors, including practices related to child care at early days of life, are known to affect child mortality [17, 18] but how these factors relate to neonatal mortality has been rarely studied. Higher risk of neonatal mortality for rural areas compared to urban areas may reflect the distribution of antenatal healthcare services, both in terms of quality and access, between the two settings similar to those found in other studies [19, 21]. Disparities between antennal care utilisation also exist in Ghana [14, 22]. Further, although antenatal coverage is high in Ghana (97%), health facility delivery is less (74%), particularly, in rural communities. Sixty percent of women in rural areas deliver in health facilities compared to 90% of those in urban areas [22]. These factors may have contributed to the rural-urban disparities as well as the regional differences in the neonatal mortality found in this study. There are individual level characteristics, which may have specific influence on neonatal health irrespective of clustered characteristics at the community levels.

Another interesting finding in this study is that maternal obesity was associated with increased risk of neonatal death. When this relationship was investigated graphically, it was also found that the risk associated with obesity and neonatal mortality is highest during the early neonatal period. This finding is consistent with those found in Sub-Saharan population-based study [23]. Obesity co-morbid with other risks such as gestational diabetes, pre-eclampsia and hypertension all of which could affect the growth and development of the foetus and consequently affect neonatal survival. Recent investigations have reported alarming rise in obesity among women in Sub-Saharan Africa [24] as well as in Ghana [25]. Considering the rising obesity epidemic, the effect of obesity on neonatal mortality found here deserves attention. It emphasises the need for a holistic approach in accelerating the slow progress made so far in addressing child mortality in general and neonatal mortality in particular.

Wealth index, which measured the household asset of the respondents, seems to have a positive gradient with neonatal mortality. Cresswell, et al. [23] presented similar relationship. Although in this study this relationship was not statistically significant, probably due to few cases, this counterintuitive finding deserves further investigation. Children of women living in regions other than the Greater Accra region had increased risk of neonatal death. The Greater Accra region being the region which houses the capital city is likely to have the most well equipped private and government health facilities for gynaecology, obstetrics and paediatric services compared to the other regions. This could explain the differences in the neonatal mortality by geographical regions.

This study investigated neonatal mortality using a nationally representative sample of three survey conducted in 2003, 2008 and 2014. All the variables used in this study were self-reported and are therefore subject to the social desirability within the communities where the women lived in the Ghanaian context in general. Also, recall bias might have been introduced since the measures for births and socio-demographic variables were assessed retrospectively, within the past 5 years preceding the study. Differential misclassification of neonatal death, particularly early neonatal death as stillbirth among the respondents could lead to spurious associations. However, evidence from previous studies, which validated such measures in retrospective and longitudinal surveys suggests that these estimates are accurate and reliable [26]. Initial assessment of the health data in the DHS-I suggests that they are accurate estimates [27]. Furthermore, a recent study of the validity of the DHS neonatal mortality measures concluded that notwithstanding the limitations of the surveys, they provide the most reliable estimates of neonatal mortality in LMICs [28]. Moreover, the use of three population-based data in this study provides sufficient power for robust estimates.

The risk of dying within the first month of life was higher for early neonates. Moreover, rural residency, birth order and maternal obesity showed higher risk for neonatal death. Likewise, children of women living in regions other than the Greater Accra region had increased risk of neonatal death. Neonatal mortality is a global public health issue and accounts for inequalities in health not only between LMICs and high income countries but also within LMICs [29]. Therefore, this study adds to our understanding regarding the time to event and the socio-demographic dynamics and  this important health outcome. The findings of this study implicate the important role of biological, environmental and geographical factors in neonatal mortality. The finding of higher risk of neonatal mortality among obese women has important implications for the emerging obesity in Ghana just as in other LMICs.

## Conclusion

The risk of neonatal mortality is highest during the first week of life and it is socio-demographically patterned. Overall, the findings emphasise the need for a holistic approach to reducing neonatal mortality in order to realise the Sustainable Development Goal 3, which is aimed at reducing neonatal mortality to 12 per 1000 live births. Future studies investigating the influence of maternal morbidities, for example, gestational diabetes, pre-eclampsia, hypertension and other chronic diseases on neonatal mortality, especially in LMICs will increase our understanding further on the risk factors of neonatal mortality. This will shed light on  effective neonatal survival interventions.

## Data Availability

The data is publicly available with permission from Measure DHS, USA.

## Abbreviations

CI: Confidence interval
GDHS: Ghana demographic and health survey
DHS: Demographic and health survey
HR: Hazard ratio
LMICs: Low- and middle-income countries
MDGs: Millennium development goals
SDGs: Sustainable development goals
